# Mechanically-induced GDF15 Secretion by Periodontal Ligament Fibroblasts Regulates Osteogenic Transcription

**DOI:** 10.1038/s41598-019-47639-x

**Published:** 2019-08-08

**Authors:** Judit Symmank, Sarah Zimmermann, Jutta Goldschmitt, Eik Schiegnitz, Michael Wolf, Heinrich Wehrbein, Collin Jacobs

**Affiliations:** 1 Polyclinic for Orthodontics, University Hospital Jena, Jena, Germany; 2 Polyclinic for Orthodontics, University Medicine Mainz, Mainz, Germany; 3 Department of Oral-, Maxilla-, Facial Surgery, University Medicine Mainz, Mainz, Germany; 4 Polyclinic for Orthodontics, University Hospital RWTH Aachen, Aachen, Germany

**Keywords:** Orthodontics, Cell biology

## Abstract

The alveolar bone provides structural support against compressive and tensile forces generated during mastication as well as during orthodontic treatment. To avoid abnormal alveolar bone resorption and tooth loss, a balanced bone turnover by bone-degrading osteoclasts and bone-generating osteoblasts is of great relevance. Unlike its contradictory role in regulating osteoclast and osteoblast cell differentiation, the TGF-β/BMP-family member GDF15 is well known for its important functions in the regulation of cell metabolism, as well as cell fate and survival in response to cellular stress. Here, we provide first evidence for a potential role of GDF15 in translating mechanical stimuli into cellular changes in immature osteoblasts. We detected enhanced levels of GDF15 *in vivo* in periodontal ligament cells after the simulation of tooth movement in rat model system as well as *in vitro* in mechanically stressed human periodontal ligament fibroblasts. Moreover, mechanical stimulation enhanced GDF15 secretion by periodontal ligament cells and the stimulation of human primary osteoblast with GDF15 *in vitro* resulted in an increased transcription of osteogenic marker genes like *RUNX2*, *osteocalcin* (*OCN)* and *alkaline phosphatase* (*ALP)*. Together, the present data emphasize for the first time a potential function of GDF15 in regulating differentiation programs of immature osteoblasts according to mechanical stimulation.

## Introduction

The continuous remodeling of the alveolar bone in response to mechanical stimulation maintains strength, prevents tissue damage and secures teeth anchorage^1^. During mastication as well as during orthodontic tooth movement (OTM) mechanical stimuli in form of tensile and compressive strains are generated^2,3^. These forces influence mechanosensitive cells of the periodontal ligament (PdL), that is closely attached to the alveolar bone, leading to changes in PdL cell vitality, proliferation and differentiation^4,5^. These changes are based on the activation of cellular signaling cascades, followed by a differential gene and protein expression^6,7^. Mechanically activated PdL cells therefore release crucial factors that modulate the initiated local inflammation together with differentiation programs of diverse cells regulating alveolar bone remodeling processes^8,9^. Among PdL cells, PdL fibroblasts show many osteoblast-like properties, as they express osteogenic markers like alkaline phosphatase and form mineralized nodules *in vitro*^10–12^. Furthermore, they also have important roles in osteoclastogenesis^12,13^ and are therefore a suitable cell culture system to study mechanical-induced changes *in vitro*^14^.

For OTM, a controlled bone resorption by osteoclasts on the compression side as well as bone formation by osteoblasts on the tensile side are of major importance for a good outcome^15^. Imbalanced bone remodeling, for example by increased bone resorption may induce alveolar bone degradation^16^ and disorders in bone metabolism leading to enhanced osteoclast activation could facilitate root resorption^16^. The severity of these risks is further reinforced by chronic periodontal inflammation as well as increased nicotine exposure^9,17^ and obesity^18^. However, the underlying cellular mechanisms are yet not fully understood. To minimize the risks, elucidating relevant key regulatory factors is of great importance and therefore in the focus of dental research.

*Growth differentiation factor* (GDF) 15 belongs to the *transforming growth factor* (TGF)-β and *bone morphogenetic protein* (BMP) superfamily^19,20^. The relevance of many members of the TGF-β/BMP protein family in regulating bone development and metabolism has already been demonstrated in numerous studies (reviewed in Chen, *et al*.^21^). Disturbances of the TGF-β/BMP system are responsible for the pathogenesis of many bone diseases and arthritis^22^. Initially detected in activated macrophages^19^, GDF15 was found rather weakly expressed in most tissues under physiological conditions^23^. However, under pathological conditions or cellular stress, GDF15 production was shown to increase dramatically^24,25^.

The diverse roles of GDF15 in the regulation of inflammatory processes, cell differentiation as well as cell repair and cell death have been described in several tissues (reviewed in Breit, *et al*.^20^). Regarding the function of GDF15 in bone metabolism, the data are to some extent contradictory supporting a GDF15-induced osteoclast activation but also a reduced osteoclast maturation upon increased GDF15 expression^26–28^. However, some studies already showed that in myocardial and trabecular meshwork cells GDF15 expression depends on mechanical stimuli^29–31^. For this reason, we asked whether GDF15 might also have important functions in alveolar bone remodeling initiated through mechanical stimulation, which typically occur during mastication and OTM.

To reveal a potential role of GDF15, we first analyzed its expression *in vivo* after simulated tooth movement in rats, as well as *in vitro* in periodontal ligament fibroblasts subjected to compressive and tensile forces. Furthermore, we examined a potential GDF15-dependent transcription of relevant osteogenic marker genes in primary osteoblasts stimulated with recombinant GDF15 protein.

## Results

### GDF15 expression is increased in periodontal ligament cells upon *in vivo* mechanical stimulation

**Figure 1:**
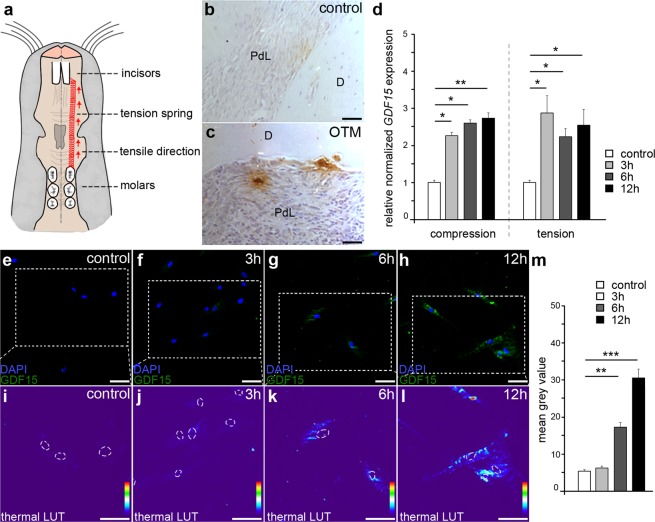
Mechanical stimulation leads to increased GDF15 expression *in vivo* and *in vitro*. (**a**) Schematic drawing of the ventral view of a rat upper jaw illustrates simulated orthodontic tooth movement (OTM) of first molars towards incisors according to Jäger *et al*.^45^. (**b**,**c**) Representative microphotographs of GDF15 expression (amber) at the border between periodontal ligament and dentin in rat upper jaw sections of control animals (**b**) and animals undergoing OTM (**c**). (**d**) Quantitative expression analysis of *GDF15* in either stretched or compressed human periodontal ligament fibroblasts (HPdLF) compared to unstimulated controls. (**e–l**) Representative microphotographs of GDF15 expression in unstimulated controls (**e**,**i**) or HPdLF stimulated for 3 h (**f, j**), 6 h (**g**,**k**) or 12 h (**h**,**l**) with biaxial tensile strain show fluorescence staining (**e**–**h**) of GDF15 (green) and nucleic staining with DAPI (blue) as well as fluorescence intensity as thermal color-code (thermal LUT; **i**–**l**). The dotted lines in e-h indicate the area of the magnified details shown in **i-l**. Nuclei are surrounded by dashed lines in (**i**–**l**). (**m**) Quantitative analysis of fluorescent signals displayed as mean grey value indicating GDF15 expression in control (n = 15), 3 h (n = 15), 6 h (n = 37) and 12 h (n = 49) stretched HPdLF. For all conditions cells from biological triplicates were analyzed. *P < 0.05; **P < 0.01; ***P < 0.001; One-Way ANOVA and post-hoc test (Tukey). n refer to the number of analyzed cells. Scale bars: 50 μm in (**b, c**), 20 μm in (**e–h**). D, dentin; PdL, periodontal ligament; OTM, orthodontic tooth movement.

To test the GDF15 expression in cells of the periodontium and to study changes due to mechanical stimulation, we simulated tooth movement *in vivo* in three-month-old wistar rats (Fig. 1a). Immunohistochemical staining of rat jaw microtome sections indeed revealed weak GDF15 expression in periodontal ligament cells under control condition in untreated rats (Fig. 1b). This expression was highly increased in PdL cells close to the cementum after nine days of OTM (Fig. 1c). Interestingly, this increased staining of GDF15 was not only detectable in PdL cell cytoplasm, but also in the extracellular matrix. Together, these data emphasize a potential role of GDF15 in periodontal cells in relation to applied mechanical forces.

### Compressive and tensile strains induce an increased expression of GDF15 in cultured HPdLF

As it was reported for other important regulators in bone remodeling and tooth movement^8,9^, we asked whether GDF15 might be exclusively expressed due to compressive or tensile forces. To this end, we performed quantitative RNA expression analysis of cultured human PdL-fibroblasts treated either with compressive or tensile strain compared to untreated controls (Fig. 1d). Under physiological conditions we found only weak expression of *GDF15*, which was already significantly increased in both, compressed and stretched HPdLF, after 3 h of mechanical stimulation (Fig. 1d). After 6 h and 12 h mechanical stimulation comparable enhanced *GDF15* levels were measured as after 3 h strain application (Fig. 1d) indicating that *GDF15* expression is to some extent independent from the duration of strain application.

Immunofluorescent stainings of GDF15 in stretched HPdLF confirmed the results we found by quantitative RNA analysis on protein level (Fig. 1e–l). Upon basic conditions, weak GDF15 was detectable, shown as false-color image (Fig. 1e) and as magnified thermal color code indicating the intensity of fluorescent staining compared to the background (Fig. 1i). With increasing duration of mechanical stimulation, we detected higher cytoplasmic GDF15 signals (Fig. 1f–h,j–l) with the highest level after 12 h of tensile strain application (Fig. 1h,l).

However, as the increase in *GDF15* transcription was not significantly different between HPdLF treated with compressive or tensile strain, we hypothesize that GDF15 has important functions on both strain sides.

### GDF15 stimulates the expression of osteogenic marker genes in primary osteoblasts

**Figure 2:**
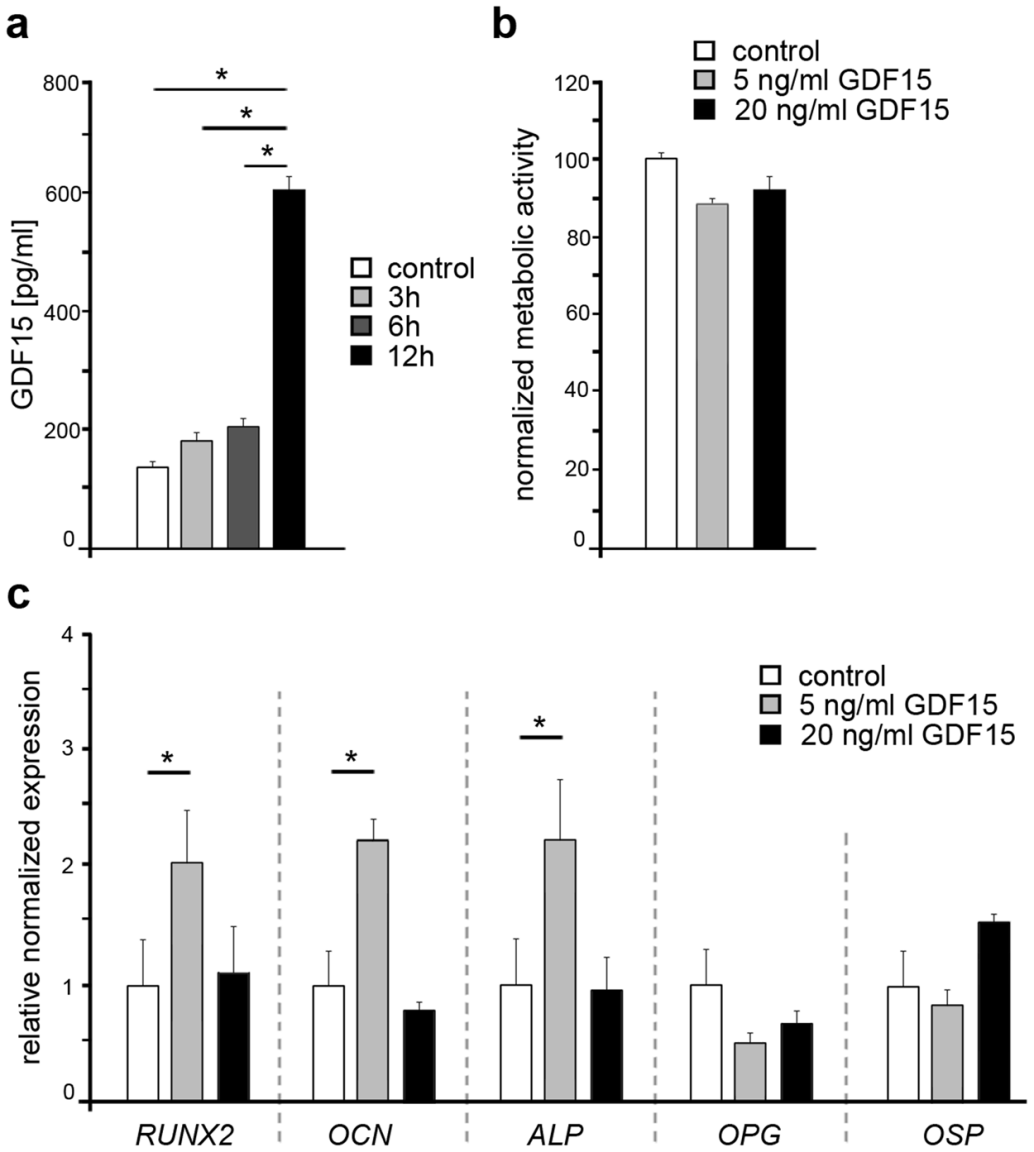
GDF15 is secreted by PdLF according to mechanical stress and stimulates osteogenic transcription in primary osteoblasts *in vitro*. (**a**) Quantitative analysis of GDF15 secretion of 3 h, 6 h or 12 h biaxial stretched HPdLF compared to unstimulated control. (**b**) Analysis of metabolic activity in human primary osteoblasts (HOB) stimulated either with 5 ng/mL or 20 ng/mL GDF15 recombinant protein compared to unstimulated control. (**c**) Quantitative expression analysis of the osteogenic marker genes *RUNX2*, *osteocalcin* (*OCN*), *alkaline phosphatase* (*ALP*), *osteoprotegerin* (*OPG*) and *osteopontin* (*OSP*) in HOB stimulated either with 5 ng/mL or 20 ng/mL recombinant GDF15 protein compared to unstimulated control. *P < 0.05; One-Way ANOVA and post-hoc test (Tukey).

As secreted members of the TGF/BMP superfamily bind to specific receptors of neighboring cells and activate intracellular signaling cascades^22^, we analyzed the release of GDF15 by HPdLF. Compared to unstimulated controls, we found a significant increase of GDF15 in cell culture supernatant of 12 h mechanically stimulated HPdLF (Fig. 2a) proving that mechanical stimulation induce GDF15 release in HPdLF.

Neither the stimulation with low (5 ng/mL) nor with high (20 ng/mL) levels of GDF15 affected the metabolic activity of cultured human primary osteoblasts (HOB), as MTT test revealed (Fig. 2b). To evaluate possible functions of GDF15 in regulating osteoblast differentiation, we further analyzed the expression of the osteogenic marker genes *RUNX2*, *osteocalcin* (*OCN*), *alkaline phosphatase* (*ALP*), *osteoprotegerin* (*OPG*) and *osteopontin* (*OSP*) in HOB stimulated with 5 ng/mL or 20 ng/mL recombinant GDF15 protein (Fig. 2c). Stimulation with low levels of GDF15 significantly increased the expression of *RUNX2*, *OCN* und *ALP*, whereas *OPG* and *OSP* levels remained unchanged. However, in contrast to low levels, we could not detect changes in HOB stimulated with high GDF15 concentrations indicating that gene expression in HOB highly depends on the dose of GDF15 for stimulation.

Altogether, our study primarily shows *in vivo* and *in vitro* that GDF15 is expressed in human periodontal ligament cells and increases alongside with the application of compression and tension strain. Moreover, secreted GDF15 stimulates the expression of the osteogenic marker genes *RUNX2*, *OCN* and *ALP* in primary human osteoblasts possibly forcing osteoblast differentiation. This suggests an important role of GDF15 in regulating relevant processes in alveolar bone remodeling activated by mechanical stimulation such as mastication or orthodontic tooth movement.

## Discussion

Alveolar bone remodeling due to applied mechanical stimuli is based on a balanced bone turnover, which is ensured by a tightly regulated bone resorption and formation by osteoclasts and osteoblasts^15^. Several key factors, facilitating bone remodeling, strongly depend on a mechanical stimulation during mastication or orthodontic treatment, either in their expression or their activity^9^. In this regard, the TGF-β/BMP protein family is involved in the integration of mechanobiological signals, which is of major importance for the skeletal development as well as for bone homeostasis in adulthood and elderly^22,32^. The expression of one family member, the growth and differentiation factor GDF15, was already been described to depend on mechanical stimuli in cardiac muscle cells^29,30^ as well as in trabecular meshwork cells^31^. Here, we could also detect for the first time enhanced GDF15 expression in periodontal ligament cells upon mechanical stimulation *in vivo* during simulated tooth movement in rats as well as *in vitro* in cultured human periodontal ligament fibroblasts. The increase in GDF15 expression was thereby comparably high in compressed and stretched cells suggesting that the type of mechanical stimulation is not of major relevance. This seems likely as both mechanical forces induce cytoskeletal alterations^33–35^, which were already shown to result in enhanced GDF15 expression in LNCaP prostate carcinoma cells^36^. Vice versa, also GDF15 has been reported to affect the expression of cytoskeletal organization genes^31^, which emphasizes a complex network around GDF15 regulating feedforward and feedback reactions upon mechanical stimulation. Cytoskeletal rearrangements in periodontal fibroblast as well as the reorganization of the surrounding extracellular matrix (ECM) are basic processes during OTM^37,38^. However, many members of the TGF-β/BMP protein family exert transcriptional changes after being secreted followed by the binding to specific receptors on neighboring cells and activation of downstream cascades^22,32^.

GDF15 is released by diverse cells due to metabolic, chemical and mechanical stress^20,24–28^. We also detected significantly higher GDF15 levels in the media of stretched PdLF compared to unstressed controls. Beside the release of matured GDF15 protein, GDF15 is partially secreted as unprocessed propeptide bound to ECM components^25^ and extracellular N-terminal cleavage by the matrix metalloproteinases (MMP) produce the active form of the GDF15 protein^39,40^. Following orthodontic treatment, we also detected increased staining of GDF15 in the ECM of rat jaw tissue. Due to the lack of antibody specificity, we could not distinguish between the GDF15 propetide and the matured state. Nevertheless, as MMP’s are highly expressed and activated in regard to mechanical stress^41^, one could speculate if extracellular bound GDF15 propeptide functions as a fast activator for further processes during alveolar bone remodeling like the initiation of cell differentiation.

Due to their local proximity and direct cell contacts, PdL cells contribute to the regulation of osteogenic differentiation and maturation programs^42^. TGF-β/BMP proteins are well known for their diverse roles in driving osteoblast, but also osteoclast development^22^. However, the functions of GDF15 are described in several studies somehow contradictory. *In vitro* studies by Vanhara^26^ on murine mononuclear bone marrow cells and RAW264.7 macrophages showed that GDF15 inhibits the formation of mature osteoclasts in a dose-dependent manner. In contrast to that, Hinoi^27^ demonstrated *in vitro* and *in vivo* that GDF15 released by osteocytes supports the differentiation of murine osteoclasts. This statement was confirmed by *in vitro* studies on human cell lines^28^. In addition, the expression of osteoblast-specific markers such as *RUNX2* and *OCN* as well as the ALP activity were significantly reduced in human mesenchymal bone marrow stem cells after stimulation with GDF15 indicating a reduced osteoblastic differentiation^28^. In contradistinction to these results, we found a significantly increased expression of osteogenic marker genes like *RUNX2*, *OCN* and *ALP* in GDF15-stimulated primary osteoblasts. Although we could identify changes in the transcription profile of primary osteoblast in relation to GDF15 stimulation, this does not necessarily correlate with changes in protein expression^43^. This makes further investigation of protein levels of osteogenic markers and differentiation analysis feasible. However, to what extent the higher multipotency of mesenchymal bone marrow stem cells compared to primary osteoblasts isolated from femoral trabecular bone tissue drive GDF15-induced expressional changes also needs further investigation. This somehow leads to one further limitation this study regarding the transfer of data from primary osteoblasts isolated from femoral trabecular bone tissue to osteoblasts of the PdL and alveolar bone, which potentially show differences in their differentiation program.

Nevertheless, GDF15-induced osteogenesis at the tension side would be favorable during OTM and probably depends on a complex interplay of diverse regulatory proteins. Further studies, for example on PdL cells and osteoblasts isolated from orthodontic patients could clarify the specific role of GDF15 in this regulatory network.

All together, we provide strong evidence for an important function of GDF15 in modulating relevant processes like osteoblast differentiation during alveolar bone remodeling in relation to mechanical forces generated during mastication and orthodontic tooth movement. Due to its stress-induced expression and the diverse roles in regulating cell function, development and survival, GDF15 could also be of great interest for treating problems like increase bone or root resorptions. For dental and orthodontic research, results of this study provide an excellent basis for further studies elucidating the mechanism of GDF15-dependent cellular changes.

## Material and Methods

### Animals

All animal procedures were approved by the local government (LANUV; North Rhine-Westphalia; Germany) and performed in strict compliance with the EU directives 86/609/EWG and 2007/526/EG guidelines for animal experiments. Animal care and handling as well as the experimental study were in accordance with all international, national and local guidelines. Animals were housed in plastic cages under 12-h light/dark conditions with ad libitum access to food and water. NC3Rs ARRIVE guidelines^44^ were followed.

### Orthodontic tooth movement (OTM) in rats

Orthodontic tooth movement was performed on ten 3-month-old male wistar rats (Charles River Laboratories). As control group 3-month-old male wistar rats (Charles River Laboratories) were used. For *in vivo* simulation of OTM, the established model of Jäger *et al*.^45^ based on the method of Ong *et al*.^46^ was applied. For the insertion of the orthodontic appliance between the maxillary right first molar and the incisors Rompun® (Bayer) and Ketavet® (Pharmacia and Upjohn) were applied for anaesthesia. Stretched closed coil strings of 0.012 inch nickel-titanium wires were used with a force of 0.5 N. To avoid application loss the mesial and distal surfaces of molars and incisors were notched. Additionally, shortening of the incisors was performed to avoid deformation. Mesial movement of the first molars towards the incisors was performed for nine days and compared to untreated animals. Animal weights were controlled before and after treatment. Feeding behavior was controlled daily.

For preparation of tissue samples, rats were killed by cervical dislocation, upper jaw samples were fixed in 4% formalin and embedded in paraffin prior microtome slicing. For further analysis 5 µm sagittal sections of the upper jaw were made until the central root area was reached. Three sections of three animals each were used for immunohistochemical stainings.

### Cell culture

Human periodontal ligament fibroblasts (HPdLF, Lonza) and human primary osteoblasts (HOB, PromoCell) were cultured in Dulbecco’s modified Eagle’s medium (DMEM; Invitrogen) containing 10% FBS (Life Technology), 1% L-glutamine (Thermo Fisher Scientific), 1% penicillin/streptomycin/neomycin (Sigma Aldrich), 1% L-ascorbic acid (Sigma Aldrich) and 20 µg/mL dexamethasone (Sigma Adlrich) at 37 °C, 5% CO_2_ and 95% humidity. The starvation medium contained reduced FBS concentration (1%). When reaching confluence, cells were passaged by application of Accutase® solution (Sigma Aldrich). For experiments, cells were used at passages four to six.

### Mechanical strain devices

For application of 3 h, 6 h and 12 h compressive strain, 1.0 × 10^5^ HPdLF were seeded randomly on 6-well cell-culture plates and a controlled compressive force of 2 g/cm^2^ via the application of sterilized glass plates was applied when reaching 70% confluence according to Kirschneck *et al*.^47^. For application of tensile strain, 1.0 × 10^5^ cells were cultured in pronectin-coated Bioflex® plates (Flexcell® International Corporation) for 24 h before medium was changed to starvation medium. Biaxial tensile strain was applied with 5% elongation using Flexcell® FX-3000™ Tension System (Flexcell® International Corporation).

### Stimulation with GDF15

For evaluation of GDF15-induced effects in HOBs, 1.0 × 10^5^ cells were incubated with 5 ng/mL or 20 ng/mL recombinant human GDF15 protein (R&D Systems) for 48 h followed by either RNA isolation or MTT analysis.

### RNA extraction and quantitative reverse transcription (qRT)-PCR

For gene expression analysis of HOB and stretched HPdLF, mRNA was isolated using Total RNA KIT peqGold (PEQLAB) including DNAse treatment according to manufacturer’s guidelines. RNA quality was analyzed using NanoDrop ND-1000 (PEQLAB). For cDNA synthesis, iScript cDNA Synthesis Kit (BioRad Laboratories) was used according to manufacturer’s protocol and stored at −20 °C until usage. Quantitative RT-PCR was performed with SYBR Green Supermix (BioRad Laboratories) and analyzed with the IQ5-I-Cycler (BioRad Laboratories). According to manufacturer’s protocol each qPCR consisted of following steps: denaturation at 95 °C, annealing at 56 °C and elongation at 71 °C, which were repeated 40 times followed by a melting curve analysis (start temperature 60 °C, end temperature 95 °C, increment ∆T was set to 1).

For analysis of compressed HPdLF, cells were isolated with TRIzol™ Reagent (Thermo Fisher Scientific) and centrifuged for 30 min at 13000 × g and 4 °C. The upper aqueous phase was further purified with the RNA Clean & Concentrator-5 kit (Zymo Research) including DNAse treatment according to manufacturer’s protocol. Synthesis of cDNA was performed with SuperScript™ III Reverse Transcriptase (Invitrogen) according to manufacturer’s protocols. For cDNA synthesis, the amount of input RNA were set a comparable amount in all samples to ensure similar synthesis rates. The cDNA was stored at −20 °C until usage. Quantitative RT-PCR was performed with Luminaris Color HiGreen qPCR Master Mix (Thermo Fisher Scientific) according to manufacturer’s protocols and analyzed with the qTOWER3 (Analytik Jena). For each sample and each primer three technical replicates were analyzed in each qPCR run. For each sample three biological replicates were analyzed in separate qPCR runs. The program of each run included the following optimized steps: UDG pre-treatment at 50 °C for 2 min, initial denaturation at 95 °C for 10 min, denaturation at 95 °C for 15 sec as well as annealing and elongation at 60 °C for 1 min. As annealing and elongation were both performed at 60 °C, the steps were fused. The last two steps (denaturation and annealing/elongation) were repeated 40 times followed by a melting curve analysis (start temperature 60 °C, end temperature 95 °C, increment ∆T was set to 1). Primers sequence information are displayed in Table 1.

**Table 1:** qPCR primer sequences of all analyzed genes with gene symbol and NIH ID.

Gene	Gene symbol	NCBI accession number	Primer sequence
alkaline phosphatase	*ALP*	NM_000478.6	fw ACTGCAGACATTCTCAAA rev GAGTGAGTGAGTGAGCA
actin beta	*ACTB*	NM_001101.5	fw GGAGCAATGATCTTGATC rev CCTTCCTGGGCATGGAG
glyceraldehyde-3-phosphate dehydrogenase	*GAPDH*	NM_001289745.2	fw AAAAACCTGCCAAATATGAT rev CAGTGAGGGTCTCTCTCTTC
growth differentiation factor 15	*GDF15*	NM_004864.4	fw1 GTTAGCCAAAGACTGCCACTG fw2 CCGAAGACTCCAGATTCCGA rev2 CCCGAGAGATACGCAGGTG rev1 CCTTGAGCCCATTCCACA
bone gamma- carboxyglutamate protein	*BGLAP* (alias *OCN*)	NM_199173.6	fw CAGGCGCTACCTGTATCA rew CTGGAGTTTATTTGGGAG
TNF receptor superfamily member 11b	*TNFRSF11B* (alias *OPG*)	NM_002546.4	fw GAAGGGCGCTACCTTGA rev GCAAACTGTATTTCGCTC
RUNX family transcription factor 2	*RUNX2*	NM_001015051.3	fw CCCACGAATGCACTATCC rev GGACATACCGAGGGACA
ribosomal protein L22	*RPL22*	NM_000983.4	fw TGATTGCACCCACCCTGTAG rev GGTTCCCAGCTTTTCCGTTC
TATA-box binding protein	*TBP*	NM_003194.5	fw CGGCTGTTTAACTTCGCTTCC rev TGGGTTATCTTCACACGCCAAG

Primer sequences were ifndicated in 5′-3′ direction with fw as forward and rev as reverse.

Primer design was performed with Primer3 (web version 4.1.0)^48^. To minimize the risks of hairpin structures and dimers, primers were tested with BeaconDesigner™ Free Edition (Premier BioSoft International). With BLAST® (U.S. National Library of Medicine) *in silico* specificity of the newly designed primers was verified. For revealing primer specificity, melting curve analysis and agarose gel electrophoresis was performed. Primer amplification efficiency was tested and ranged from 98.4–102.3%.

*GAPDH* and *ACTB* were used as housekeeping genes for normalisation in HOBs and stretched HPdLF as these analyses were performed earlier than the ones in compressed HPdLF. According to Kirschneck *et al*.^47^ *TBP* and *RPL22* were used in compressed hPdLF as housekeeping genes. Data were analyzed with ΔΔCT method^49^.

### Immunocytochemistry and immunhistochemistry

For detection of GDF15 expression in stretched HPdLF, cells were first isolated with Accutase® solution (Sigma Aldrich) from Bioflex® plates and seeded on coverslips. After 24 h cultivation, cells were then fixed in methanol/aceton (1:1) for 10 min at − 20 °C. Blocking of unspecific binding sites was performed with 0.25% Casein/0.1% BSA for 30 min at room temperature. Primary antibody incubation targeting GDF15 (1:50, Santa Cruz) occurred over night at room temperature. Alexa 594-tagged secondary antibody (1:50; Lifetechnologies) was applied for 3 h for visualization and nuclei were stained with DAPI (2 µg/mL, Sigma-Aldrich) for 5 min at room temperature. Three biological replicates were stained for each condition.

For detection of GDF15 after OTM in rats, 5 µm microtome slices of rat upper jaws were used that showed the border between periodontal ligament and dentin. Primary antibody incubation (GDF15, 1:200; R&D Systems) was performed for 20 h in TBS/BSA at 4 °C followed by the detection with a HRP-labeled secondary antibody (1:1000; DAKO) for 30 min at room temperature. Tissue sections were stained in a 3,3‘-diaminobenzidine solution (Sigma Chemicals Co, USA), counterstained with Mayer’s haematoxylin, dehydrated and cover slipped for light microscopical analysis.

### Enzyme-linked immunosorbent assay

Quantification of GDF15 levels in cell culture supernatant of stretched and control HPdLF was performed 24 h after stimulation with Human GDF15 DuoSet ELISA Kit (R&D Systems) according to manufacturer’s protocols. The analysis was performed with three biological replicates.

### MTT cell vitality test

Cell vitality of HOB incubated with either 5 ng/mL or 20 ng/mL recombinant human GDF15 protein (R&D Systems) for 48 h was analyzed with MTT colorimetric assay (Sigma Aldrich) according to manufacturer’s protocol. The analysis was performed with three biological replicates.

### Microscopy, image analysis and statistics

Images were either taken with the fluorescence microscope BZ-9000 (Keyence) or the Carl Zeiss Axioscope. For quantitative analysis of immunofluorescence, immunostaining of all slices was performed simultaneously and image acquisition was performed with identical microscope settings. Grey values of GDF15 fluorescent signals were measured with Fiji software^50^ for each cell and background correction was performed by subtracting the mean of three independent grey value measurements of cell surrounding areas. Figure illustration was performed with Adobe Photoshop® CS5. Data are presented as the mean ± s.e.m. SPSS 21.0 (IBM-SPSS) was used for statistical analysis. One-Way ANOVA and post-hoc test (Tukey) was used to identify significant differences in gene expression levels as well as immunofluorescent signals intensity (analyzed as grey values). If not stated differently, three independent experiments were performed. n refer to the number of analyzed cells.

## Data Availability

The datasets generated during and/or analysed during the current study are available from the corresponding author on reasonable request.
